# Hydrothermal synthesis of ZnO quantum dot/KNb_3_O_8_ nanosheet photocatalysts for reducing carbon dioxide to methanol

**DOI:** 10.3762/bjnano.8.226

**Published:** 2017-10-30

**Authors:** Xiao Shao, Weiyue Xin, Xiaohong Yin

**Affiliations:** 1 School of Chemistry and Chemical Engineering, Tianjin University of Technology, Tianjin 300384, China; 2 Tianjin Key Laboratory of Organic Solar Cells and Photochemical Conversion, Tianjin University of Technology, Tianjin 300384, China; 3 School of Chemical Engineering, East China University of Science and Technology, Shanghai 200237, China

**Keywords:** CO_2_ reduction, KNb_3_O_8_ nanosheets, methanol production, photocatalysis, ZnO quantum dots

## Abstract

ZnO quantum dots and KNb_3_O_8_ nanosheets were synthesized by a two-step hydrothermal method for the photocatalytic reduction of CO_2_ to methanol where isopropanol is simultaneously oxidized to acetone . The as-prepared photocatalysts were characterized by X-ray diffraction (XRD), energy dispersive X-ray spectroscopy (EDS), scanning electron microscopy (SEM), high-resolution transmission electron microscopy (HRTEM) and UV–vis absorption spectroscopy (UV–vis). The photocatalytic activity of the materials was evaluated by formation rate of methanol under UV light irradiation at ambient temperature and pressure. The methanol formation rate of pure KNb_3_O_8_ nanosheets was found to be 1257.21 μmol/g/h, and after deposition of 2 wt % ZnO quantum dots on the surface of KNb_3_O_8_ nanosheets, the methanol production rate was found to increase to 1539.77 μmol/g/h. Thus, the ZnO quantum dots obviously prompted separation of charge carriers, which was explained by a proposed mechanism for this photocatalytic reaction.

## Introduction

In recent years, the rapidly increasing rate of carbon dioxide emissions into the global atmosphere has received significant attention as it is considered to be the primary greenhouse gas that has led to global warming and even threatens the existence and development of human society [1]. Under the pressure of severe environmental issues, researchers are seeking solutions to reduce CO_2_ emissions or to utilize the emitted CO_2_. Among various problem-solving approaches, photocatalytic reduction of CO_2_ is most promising for converting CO_2_ into useful organics without any footprint, similar to what occurs in natural photosynthesis. Until now, the organics produced by such artificial photosynthesis include methane [2], formaldehyde [3], methanol [4], methyl formate [5], among others.

Alkaline niobates, which are great potential photocatalysts, have been developed in virtue of their excellent nonlinear optical, piezoelectric, ferroelectric, ionic conductivity, selective-ion exchange and photocatalytic properties [6–9]. Zhang et al. prepared K_4_Nb_6_O_17_ with a sheet-like nanostructure by hydrothermal synthesis and found its photocatalytic activity for degrading acidic red G to be much higher than that of the commercial Degussa P25 [10]. Zhou et al. synthesized porous K_4_Nb_6_O_17_ microspheres with large surface area via a homogeneous precipitation method, which showed not only two times higher photocatalytic activity than that of the commercial Degussa P25 (the titanium dioxide particle diameter is about 25 nm) in the degradation of rhodamine B, but also a higher generating rate of H_2_ at 3.0 mmol/h under UV-light irradiation [11]. Song prepared nanocrystals of ferroelectric NaNbO_3_ phase by a hydrothermal method using Nb_2_O_5_ and NaOH as precursors in one step, where a lower concentration of Nb_2_O_5_ was favorable to form NaNbO_3_ nanorods and nanoplates, while a lower concentration of NaOH facilitated formation of NaNbO_3_ cubes [12]. Shi and his co-workers synthesized polymeric g-C_3_N_4_ coupled with NaNbO_3_ nanowires for enhancing photocatalytic reduction of CO_2_ into renewable fuel [13]. KNb_3_O_8_, a well-known layered niobate, has an orthorhombic crystal structure that consists of negatively charged sheets and is linked by NbO_6_ octahedral units and K^+^ ions between the sheets [14]. Its unique microstructure has aroused researchers' interest. Traditionally, KNb_3_O_8_ was synthesized by a solid-state reaction or alkoxide methods at more than 800 °C [15–17], which make the particle size uncontrollable. It is well-known that the morphology, size and crystal structure of the photocatalyst are crucial properties to control its photocatalytic activity [18]. Francesco and his team [19] synthesized rod-like KNb_3_O_8_ by using KCl, K_2_CO_3_, and Nb_2_O_5_ by calcination at 800 °C for 3 h. Zhan et al. [20] prepared the single-crystalline KNb_3_O_8_ nanobelts via a molten salt method and investigated its photocatalytic performance in degradation of methyl orange under UV irradiation.

To the best of our knowledge, there have been few reports about the photocatalytic reduction of CO_2_ using KNb_3_O_8_ nanosheets. In this paper, we describe the synthesis of composite photocatalysts comprised of ZnO quantum dots and KNb_3_O_8_ nanosheets, produced by hydrothermal synthesis. The as-prepared photocatalysts were tested by reducing CO_2_ in isopropanol to methanol under UV-light irradiation.

## Experimental

### Catalyst synthesis

Niobium oxide (Nb_2_O_5_, 99.99 wt %) was purchased from Aladdin Industrial Corporation. Potassium hydroxide (KOH, 96 wt %), isopropanol (C_3_H_8_O, 99.9 wt %), hydrochloric acid (HCl, 36.5 wt %), zinc chloride (ZnCl_2_, 98 wt %), sodium hydroxide (NaOH, 96 wt %) and tetraethylene glycol (TEG, 99 wt %) were purchased from Tianjin Guangfu Chemical Reagent Company. All chemicals used in this experiment were of analytical grade and used without any further purification.

ZnO quantum dots were loaded onto KNb_3_O_8_ nanosheets by a two-step hydrothermal method. Typically, 0.2 g of Nb_2_O_5_ powder in 40 mL of aqueous alkaline solution containing 1–4 mol/L KOH were heated to 180 °C for 48 h in a 75 mL sealed Teflon-lined autoclave [21]. After cooling to room temperature naturally, a clear solution was obtained. The pH of the solution was gradual adjusted with diluted HCl solution to 5–6 under stirring and a white slurry was obtained. A given amount of obtained ZnO quantum dots [22] were added into this white slurry under magnetic stirring. Then the slurry was poured into a 75 mL Teflon-lined autoclave and heated to 200 °C and kept for 48 h, where the precipitate was collected by centrifuging the mixture, then washed with ethanol and deionized water three times, and finally dried at 65 °C for 12 h for further characterization.

### Catalyst characterization

The crystal structure of the synthesized materials were characterized with a powder X-ray diffraction instrument (XRD, D/Max–2500, Rigaku). The morphology and microstructure were examined by scanning electron microscopy (FE-SEM, JEOL–JSM 700F). The chemical composition was characterized using energy dispersive X-ray spectroscopy (EDS). TEM and HRTEM images were obtained with a Tecnai G2 F20 instrument. The UV–vis diffuse reflectance spectra were captured by a Shimadzu UV–2550 UV–vis spectrophotometer using BaSO_4_ as a reference in the wavelength region of 200–800 nm. Photoluminescence (PL) spectra were measured at room temperature on a Renishaw 1000 Raman system using a 325 nm laser.

### Photocatalytic reaction

The photocatalytic reduction of CO_2_ to methanol was carried out at ambient conditions in a quartz container and batch operated slurry reactor with cooling jacket. 20 mg of the catalyst was added to 20 mL of isopropanol under magnetic stirring. High purity CO_2_ was bubbled through the isopropanol solution at a rate of 200 mL/min for 30 min to eliminate air and to saturate the solution before irradiation. Then the reactor was illuminated by a 250 W high-pressure mercury lamp with peak wavelength of 365 nm for 10 h. After the reaction, the transparent solution was centrifugally separated from the slurry and analyzed by a gas chromatograph (SCION 456–GC). The reference experiments were carried out in the absence of photocatalyst or laser irradiation, and there were no products formed.

## Results and Discussion

### Catalyst characterization

**Figure 1:**
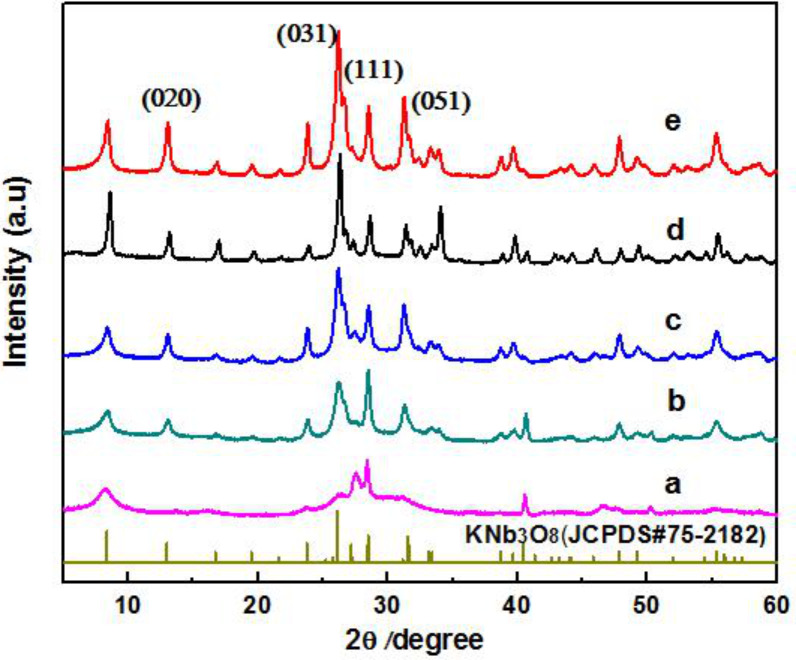
X-ray diffraction patterns of samples prepared at different KOH concentrations: (a) 0.5 M; (b) 1.0 M; (c) 2.0 M; (d) 3.0 M; (e) 4.0 M.

The two-step hydrothermal process for synthesizing KNb_3_O_8_ nanosheets was influenced by several factors. Among them, the concentration of the KOH solution, which is presented by the pH, is the critical factor [23]. Figure 1 shows the powder XRD patterns of the as-synthesized particles with different KOH concentrations. The Nb_2_O_5_ could not completely convert to KNb_3_O_8_ under lower KOH concentration during the hydrothermal treatment [24]. Only when the KOH concentration was increased to 2 mol/L was a pure KNb_3_O_8_ phase obtained. All diffraction peaks of the samples can be readily compared to JCPDS#75-2182, thus no impurity peaks were found. With an increase in the concentration of KOH, the diffraction peaks become gradually strong. A large amount of nanosheets with length larger than 1 μm, ≈700 nm in width and <10 nm thickness were obtained.

**Figure 2:**
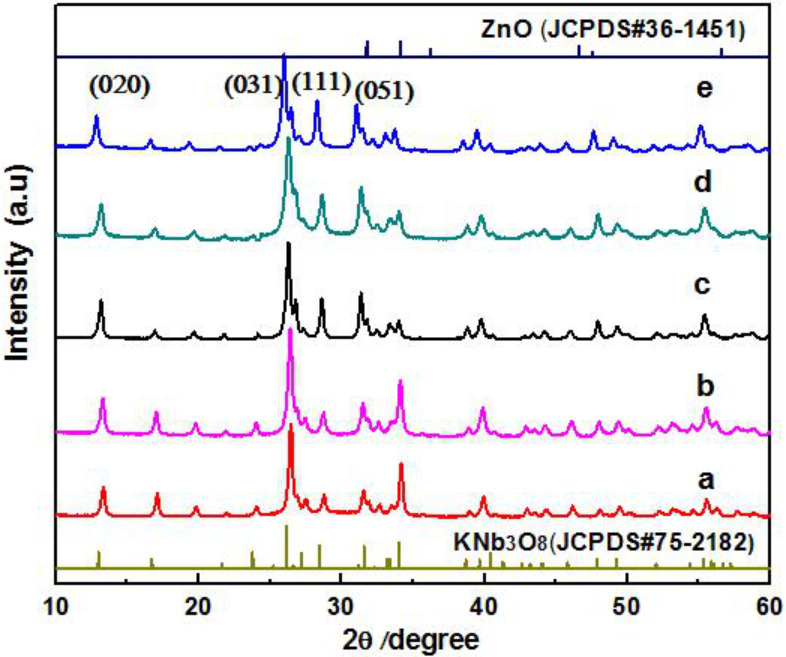
X-ray diffraction patterns of catalysts loaded with different amounts of ZnO quantum dots: (a) pure KNb_3_O_8_; (b) 1 wt % ZnO–KNb_3_O_8_; (c) 2 wt % ZnO–KNb_3_O_8_; (d) 3 wt % ZnO–KNb_3_O_8_; (e) 4 wt % ZnO–KNb_3_O_8_.

The XRD patterns of different amounts of ZnO quantum dots loaded onto KNb_3_O_8_ nanosheets are shown in Figure 2, where the diffraction peaks of pure KNb_3_O_8_ at 2θ of 12.98°, 26.62°, 28.48°, and 31.62° correspond to the (020), (031), (111) and (051) crystal planes of the perovskite KNb_3_O_8_ (JCPDS #75-2182). When the ZnO quantum dots are loaded onto the KNb_3_O_8_, the XRD patterns of the composites seemed not to change, which illustrates that the ZnO quantum dots did not enter the frame of the crystalline KNb_3_O_8_ and a small amount of ZnO was also not found.

**Figure 3:**
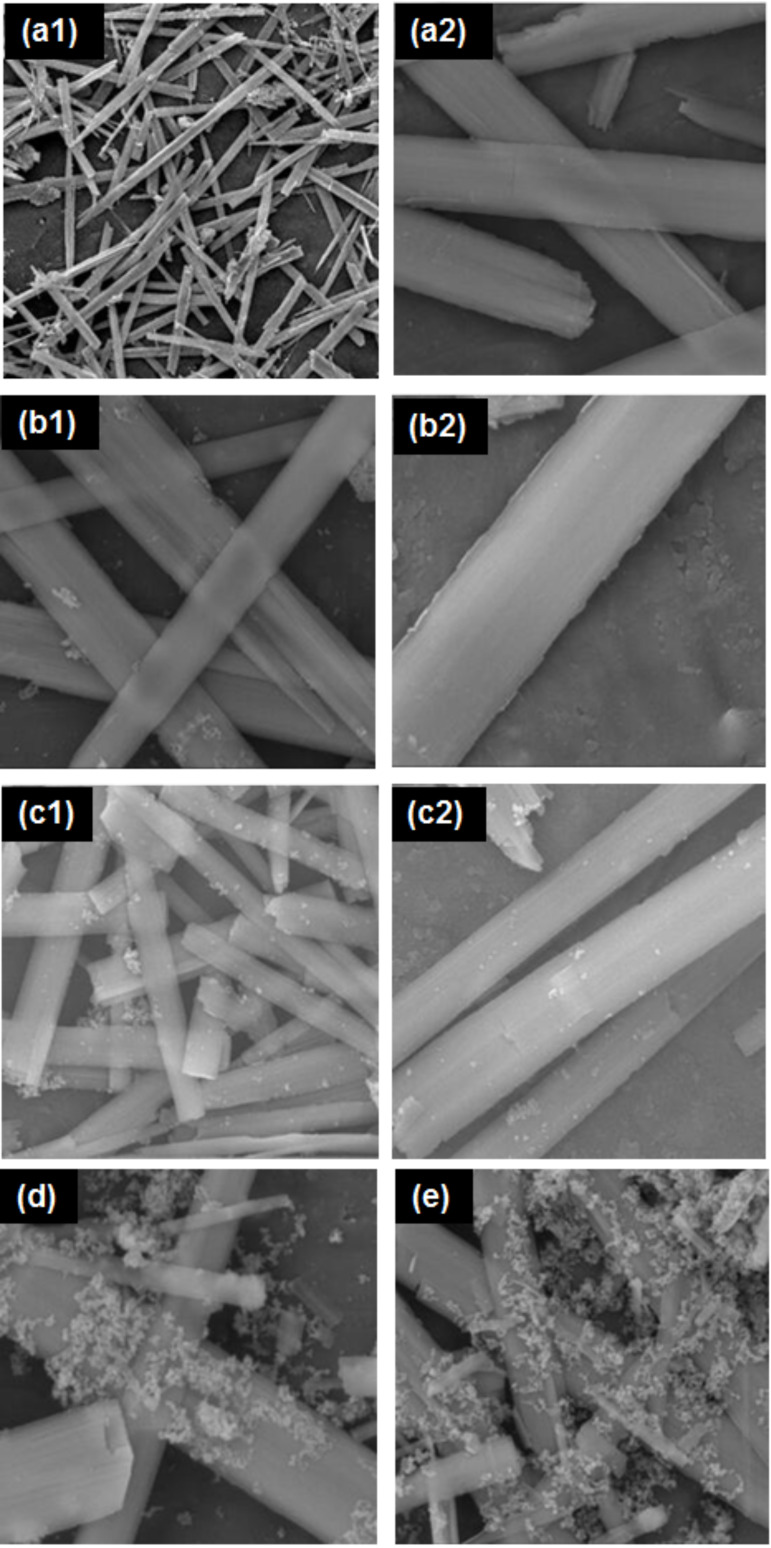
SEM images (some with higher resolution) of different ZnO loadings on KNb_3_O_8_ nanosheets: (a) 0 wt %; (b) 1 wt %; (c) 2 wt %; (d) 3 wt %; (e) 4 wt %.

The morphology of KNb_3_O_8_ nanosheets loaded with different amounts of ZnO quantum dots was characterized by SEM and is shown in Figure 3. The morphology of the as-synthesized KNb_3_O_8_ material is shown in the Figure 3a,b. The ZnO quantum dots were found on the surface of the KNb_3_O_8_ nanosheets. The higher loadings of ZnO quantum dots caused aggregation as shown in Figure 3d,e.

**Figure 4:**
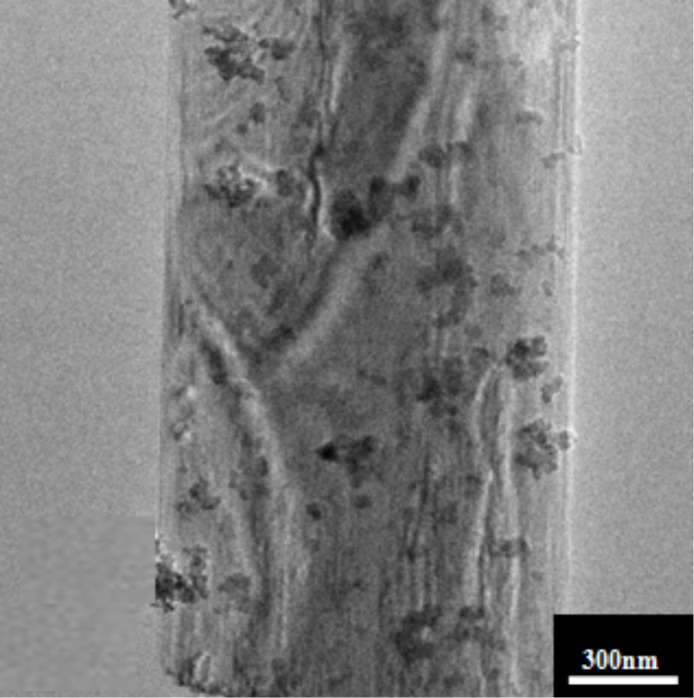
TEM image of the 2 wt % ZnO quantum dot/KNb_3_O_8_ nanosheet composite photocatalyst.

**Figure 5:**
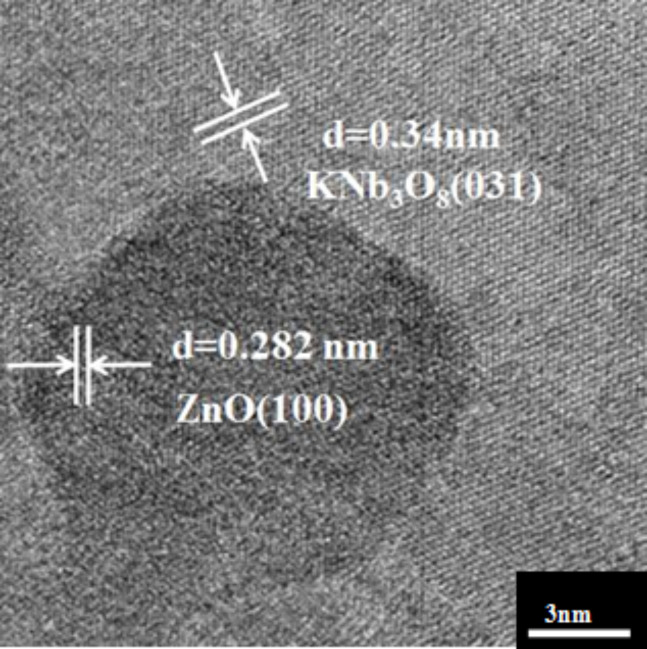
HRTEM image of the 2 wt % ZnO quantum dot/KNb_3_O_8_ nanosheet composite photocatalyst.

**Figure 6:**
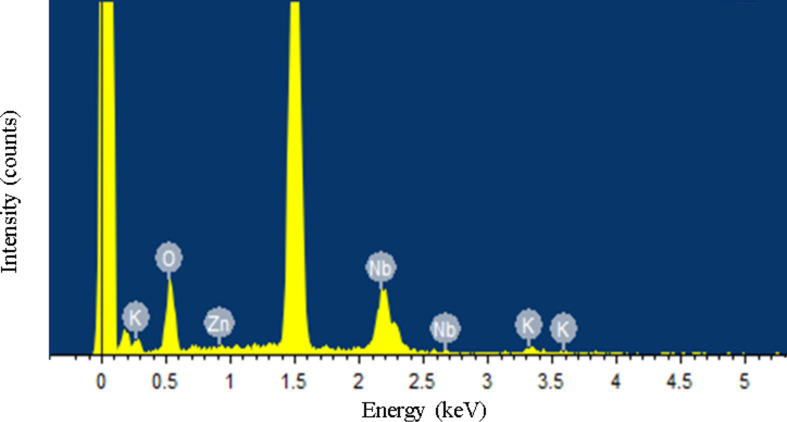
Energy dispersive X-ray spectroscopy pattern of the 2 wt % ZnO quantum dot/KNb_3_O_8_ nanosheet composite photocatalyst.

The crystallinity and morphology of the 2 wt % ZnO quantum dots loaded onto KNb_3_O_8_ nanosheets was characterized via TEM (Figure 4) and HRTEM (Figure 5). The crystalline ZnO quantum dot average diameter was about 10 nm and they were found dispersed on the surface of the KNb_3_O_8_ nanosheets. The HRTEM image revealed a lattice spacing of 0.282 nm (Figure 5), which clearly matches the lattice spacing of the (100) plane of the ZnO [25]. The lattice fringe spacing of 0.34 nm was observed in Figure 5, which is ascribed to the (031) plane of KNb_3_O_8_. Additionally, the elemental EDS analysis showed the sample to be composed of K, Nb, Zn and O in Figure 6.

**Figure 7:**
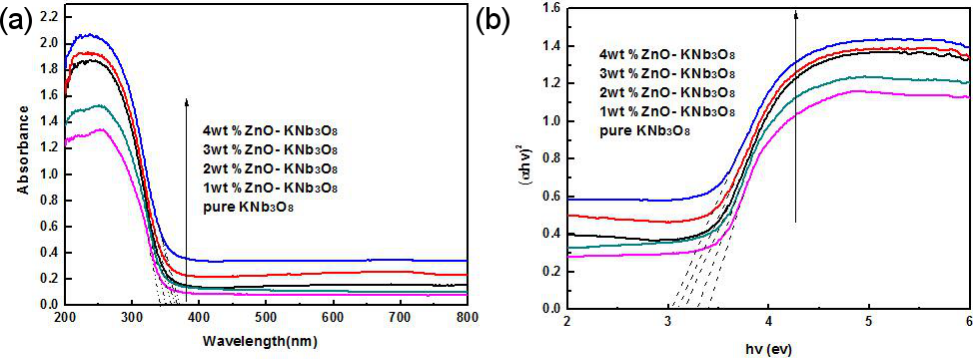
(a) UV–vis diffuse reflectance spectra of the ZnO quantum dot/KNb_3_O_8_ nanosheet composite photocatalysts; (b) (α*hv*)^1/2^ versus the photon energy of the ZnO quantum dot/KNb_3_O_8_ nanosheet composite photocatalysts.

The UV–vis diffuse reflectance spectra for different amounts of ZnO quantum dots loaded onto KNb_3_O_8_ nanosheets are illustrated in Figure 7. The steep absorption edge of the pure KNb_3_O_8_ nanosheets occurred around 343 nm under UV light owing to its large intrinsic band gap. When increasing the ZnO quantum dot loading, the sample displayed an absorption threshold at 376 nm. Compared with the pure KNb_3_O_8_ nanosheets, we found a significant red-shift of the absorption edge, which could attribute to the enhancement observed by the ZnO quantum dots upon visible-light absorption. The optical band gap (*E*_g_) of semiconductors could be estimated from the Kublka–Munk transformation. The bang gap value of pure KNb_3_O_8_ nanosheets was estimated to be 3.46 eV, which is in agreement with the previous reports [26]. After the ZnO quantum dots were loaded, the bang gap value reduced to 3.12 ev.

### Photocatalytic reduction of CO_2_

**Figure 8:**
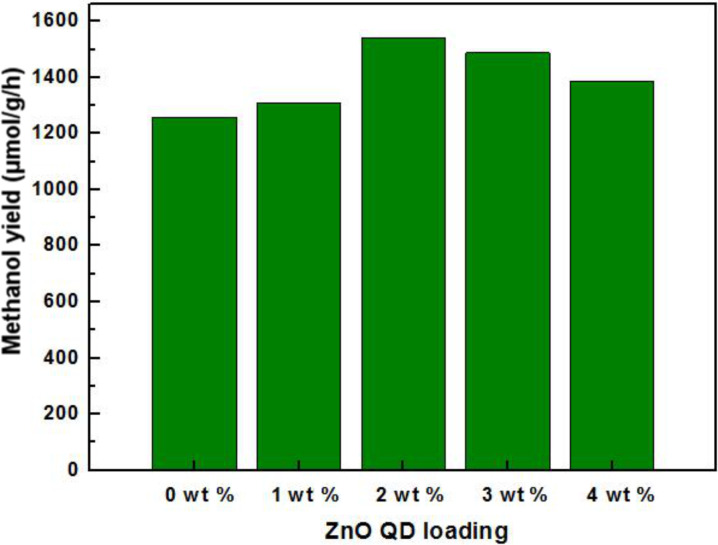
Yield of methanol using KNb_3_O_8_ nanosheets with different amounts of ZnO quantum dot (QD) loadings: (0) pure KNb_3_O_8_; (1) 1 wt % ZnO QDs; (2) 2 wt % ZnO QDs; (3) 3 wt % ZnO QDs; (4) 4 wt % ZnO QDs.

The photocatalytic activity of the as-synthesized samples towards the photocatalytic reduction of CO_2_ in isopropanol is shown in Figure 8. The pure KNb_3_O_8_ nanosheets demonstrated the lowest yield of methanol, which may be due to the unique structure of the distorted NbO_6_ octahedron and the large external surface area (37.639 m^2^/g ) of the nanosheets. Compared with pure KNb_3_O_8_ nanosheets, the yield of methanol was improved until a ZnO concentration of 2 wt % was reached. The highest yield of methanol was 1539.77 μmol/g/h for the 2 wt % ZnO quantum dots. This high yield can be ascribed to the heterojunction between the KNb_3_O_8_ nanosheets and the ZnO quantum dots, inhibiting the efficient recombination of photogenerated electron–hole pairs. However, with a superfluous amount of ZnO quantum dots, the photocatalytic activity became worse due to the aggregation of ZnO quantum dots which gradually form a recombination center for photogenerated electron–hole pairs.

**Figure 9:**
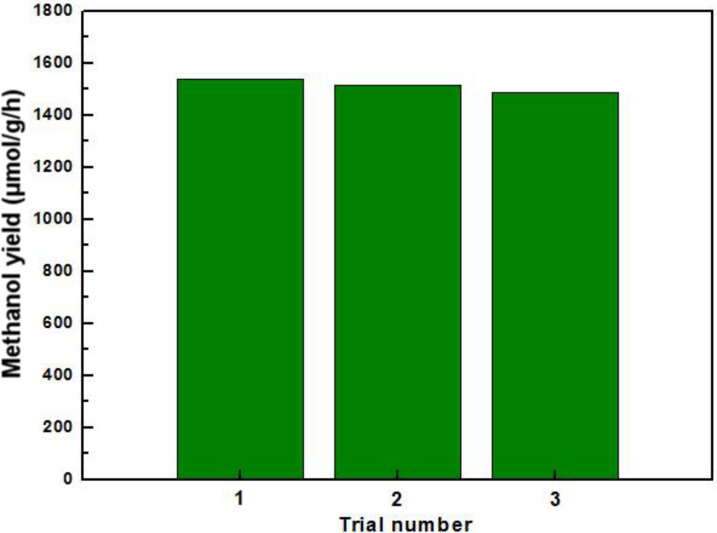
Trials showing the recyclability of the 2 wt % ZnO quantum dot/KNb_3_O_8_ nanosheet composite photocatalyst.

In addition, The stability of the ZnO quantum dots and KNb_3_O_8_ nanosheets after the photocatalytic reduction of CO_2_ was evaluated by reusing them after each batch without any treatment. In Figure 9, the composite photocatalysts of ZnO quantum dots and KNb_3_O_8_ nanosheets were recycled three times. This shows that the activity of the materials slowly declines but the formation rate of methanol was maintained at around 1500 μmol/g/h.

**Figure 10:**
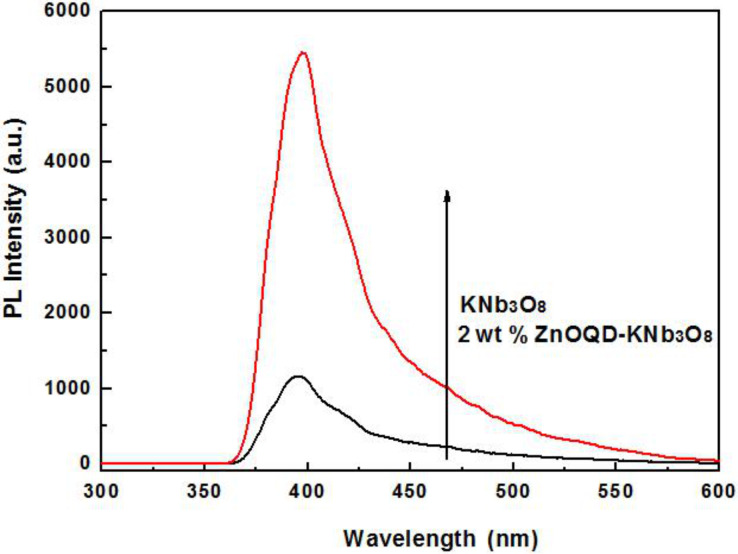
Photoluminescence (PL) spectra of the 2 wt % ZnO quantum dot/KNb_3_O_8_ nanosheet composite photocatalyst (black) compared to KNb_3_O_8_ nanosheets (red).

Photoluminescence (PL) spectroscopy was used to trace the migration and capture of photogenerated electrons and holes from the as-synthesized samples. As is known, the higher the emission peak intensity, the more efficient the recombination of photogenerated carriers, which may limit the photocatalytic performance [26]. In Figure 10, KNb_3_O_8_ nanosheets excited high emission peaks. However, after 2 wt % ZnO quantum dots were loaded onto the nanosheets, the emission intensity evidently decreased, which indicates that the photogenerated charge carriers are efficiently separated and the lifetime of free carriers is expected to be prolonged. In addition, heterostructures act as an excellent electron transport platform to facilitate the electron–hole transfer [27], efficiently enhancing the photocatalytic reduction of CO_2_.

### Reaction mechanism

**Figure 11:**
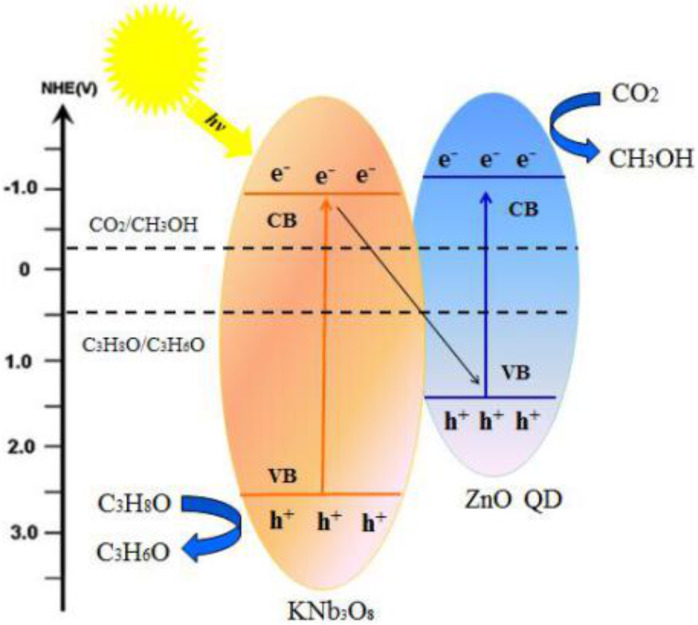
Mechanism of the photocatalytic reduction of CO_2_ to methanol on the ZnO quantum dot/KNb_3_O_8_ nanosheet composite photocatalyst.

The mechanism of photocatalytic reduction of CO_2_ to methanol on ZnO quantum dots loaded onto KNb_3_O_8_ nanosheets under UV irradiation is shown in Figure 11. Under UV radiation, the catalyst can create photogenerated electron–hole pairs. The conduction band and the valence band of the catalyst were calculated by the empirical equations [13]: *E*_CB_* = X* − *E*_c_ − 0.5*E*_g_*; E*_VB_* = E*_CB_* + E*_g_ where *X* is the absolute electronegativity of the semiconductor, *E*_c_ is the energy of the free electrons under standard hydrogen electrode potential (4.5 eV) and *E*_g_ is the band gap energy. The results indicated that the conduction band of pure ZnO quantum dots (*E*_CB_ = −1.02 V vs NHE at pH 7, the same below) [25,28] and pure KNb_3_O_8_ nanosheets (*E*_CB_ = −0.94V) are more negative than the position of the reduction of CO_2_ to methanol (*E*_CO2/CH3OH_ = −0.38 V) [29], the valence band of pure ZnO quantum dots (*E*_VB_ = 1.6 V) and pure KNb_3_O_8_ nanosheets (*E*_VB_ = 2.52 V) which are more positive than that of the isopropanol oxidation to acetone (*E*_C3H8O/C3H6O_ = 0.49 V) [30], which made the reduction of CO_2_ to methanol and the oxidation of isopropanol to acetone possible. Under UV irradiation, photoinduced electrons in the conduction band (CB) of KNb_3_O_8_ migrate to the valence band (VB) of ZnO quantum dots and combine with the holes to efficiently prevent photogenerated electron–hole pairs from vast and fast recombination. Therefore, the lifetime of free carriers is prolonged and the photocatalytic performance could be enhanced.

The photocatalytic reaction mechanism is as follows.

Photoexcitation of catalyst:


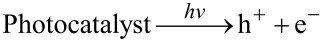


The conduction band:


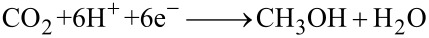


The valence band [31]:


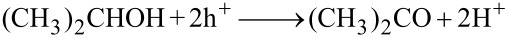


## Conclusion

In this experiment, ZnO quantum dots were loaded onto KNb_3_O_8_ nanosheets via a two-step hydrothermal method. After evaluating the as-synthesized samples for the photocatalytic reduction of CO_2_ to methanol in isopropanol under UV-light irradiation, we discovered that the methanol yield was evidently improved by the presence of ZnO quantum dots (as compared to pure KNb_3_O_8_ nanosheets). The ZnO quantum dots form a heterojunction with KNb_3_O_8_ nanosheets to efficiently separate the photogenerated electron–hole pairs and inhibit their recombination while prolonging the lifetime of free carriers. The 2 wt % ZnO quantum dots loaded onto KNb_3_O_8_ nanosheets showed superior photocatalytic activity with 1539.77 μmol/g/h of methanol yield. Furthermore, the mechanism of photocatalytic reduction of CO_2_ is photogenerated electrons and holes to be consumed for producing methanol and acetone separately.
